# No More Glass Bottles? Canned Wine and Italian Consumers

**DOI:** 10.3390/foods11081106

**Published:** 2022-04-12

**Authors:** Giordano Ruggeri, Chiara Mazzocchi, Stefano Corsi, Benedetta Ranzenigo

**Affiliations:** Department of Agricultural and Environmental Science, Production, Territory, Agroenergies, University of Milan, 20122 Milan, Italy; chiara.mazzocchi1@unimi.it (C.M.); stefano.corsi@unimi.it (S.C.); benedetta.ranzenigo@studenti.unimi.it (B.R.)

**Keywords:** alternative wine packaging, sustainable packaging, aluminium can, contingent valuation, consumer preferences, willingness to pay

## Abstract

Packaging is an important economic component of the wine industry. However, while glass bottles are the leading wine container globally, their production and handling entail severe problems in increased carbon footprint impact and waste and logistic management. As a result, the wine packaging industry has developed and commercialised several alternatives to glass bottles, including aluminium cans. However, despite producers’ efforts in proposing alternative wine packaging, there are several barriers to their diffusion, especially in countries with a long tradition of wine consumption such as Italy, and it is still uncertain if and to what degree consumers would appreciate a wine in an aluminium can. This research investigates Italian wine consumers’ preferences and willingness to pay for canned wine through a survey and the contingent valuation method. We collected data from 551 consumers regarding attitudes and preferences about their wine consumption, alternative packaging acceptance, and motivations for accepting and refusing to buy it. Only a minority of the respondents declared they would buy canned wine, while the majority would refuse for reasons related to low-quality perception and poor consideration of alternative wine packaging. The lack of knowledge is one of the main obstacles to the diffusion of canned wine. However, canned wines could address different groups of wine drinkers and consumption occasions, increasing the opportunities for winemakers, especially among non-regular consumers.

## 1. Introduction

Food packaging is one of the most dynamic and innovative components of food processing and delivery. Although necessary for adequate storage and distribution of food [1,2], packaging is a source of waste management issues and energy and resources consumption. The current debate about greenhouse gas emissions is rich and open to new solutions thanks to innovative materials and processes, also in the wine industry. According to the International Organisation of Vine and Wine (OIV) [3], 292 million hectolitres of wine were produced globally in 2018, being Italy the most important producer in terms of quantity with 54.8 million hectolitres, followed by France and Spain. With these large volumes, packaging is an important economic component of the wine industry. Since the twentieth century, when bottles replaced wooden barrels, most wine containers were made of glass, mainly for the ease of boxing and transporting them [4,5]. However, the diffusion of glass bottles as leading wine containers entails some relevant issues. 

According to Life Cycle Assessment (LCA) literature, about 45.8% of the wine carbon footprint impact comes from viticulture phases and 41.1% from bottling and packaging [1,6]. Packaging materials are responsible for 57% of the total emissions at the winery stage, with glass bottles being the dominant source (47%) [7]. The production of glass bottles contributes the most to the impact due to their weight and the massive energy consumption required for glass production [8,9,10,11]. Moreover, handling and haulage costs are higher in glass than in other materials due to its weight and fragility. Of course, glass can be recycled, and the amount of cullet used in new glass bottles can reach 95% [12], reducing the use of new materials for making bottles and emissions. However, recycling glass requires high temperatures to melt the product, which is overwhelmingly powered by fossil fuels and results in high energy consumption levels. Nevertheless, when the cullet is mixed with other materials such as plastics, metals, ceramic, mirrors, and different kinds of glass, defects in the bottles or impurities in the glass are common [4].

In recent years, the packaging industry has developed several kinds of wine packaging alternatives to glass bottles, including aluminium cans, Tetra Pack boxes, Polyethene terephthalate, and Bag in Box [1,13,14]. Among these innovations, the most successful launch in recent years is aluminium cans. From a value of less than $10 million in 2014, the US canned wine market reached over $90 million in mid-2019 [15]. As for Europe, Euromonitor estimated that the number of cans produced in Europe in 2022 will be 250 million, with a Compound Annual Growth Rate between 2012 and 2022 of 6.1% for still wines and 14.4% for sparkling wines. According to OIV elaborations of Euromonitor data, the consumption of canned wine is growing today at an annual rate of 6% in Western Europe [3]. The reasons of the success of canned wine can be summarised in some specific characteristics. Cans are suitable for preserving the quality of wine: the inner epoxy resin acts as a protective barrier between the wine and the aluminium, guaranteeing the conservation from 6 to 15 months, and they protect the wine from the light. Moreover, individual serving cans allow consumers to pair glasses of different wines with different courses and not have to open a bottle to enjoy one glass of wine [16]. Aluminium is 100% recyclable an infinite number of times, and although there are no specific studies on the wine industry, a study on LCA impacts on beer production in the UK shows that 1 l of beer packaged in glass bottles consumes 17.5 MJ of primary energy and generates 842 g of CO_2_ eq. emissions, while aluminium cans require 11.3 MJ of primary energy and emit 574 g of CO_2_ eq. [17]. According to Work [4], aluminium cans have a 67% recycle rate, a value higher than other beverage containers [18]. According to Aluminum Association, in 2015 United States consumed 88 billion cans and recycled about 56 billion of them, 64% of their volume [4]. Aluminium cans are also more compact and less fragile than glass bottles, thus requiring less packaging and transportation costs. A study from 2012 by Rexam [19], the second-largest consumer packaging company worldwide, examined the efficiency of using space transporting canned wine and CO_2_ emissions during transport, finding that CO_2_ emissions for cans are halved compared to 75 cl glass bottles. Finally, aluminium cans are more practical to carry around than glass bottles and are suitable for situations where it is inconvenient or illegal to carry glass bottles, such as on beaches, swimming pools, outdoor parties, and even while travelling on a plane.

Nevertheless, despite producers’ efforts to surpass the tradition by proposing alternative wine packaging, there are several barriers to their diffusion, especially in countries with a long tradition of wine consumption [13,20,21], and it is still uncertain if and to what degree consumers would appreciate a wine in an aluminium can. This research investigates Italian wine consumers’ preferences and WTP for canned wine through a survey and the contingent valuation method. We analyse consumers’ WTP for canned wine through a contingent valuation and investigate attitudes and preferences about wine consumption, alternative packaging acceptance, and motivations for accepting and refusing to buy it.

## 2. Wine Consumers’ Perception of Alternative Packaging

Several studies have analysed consumers’ behaviour towards environmental concerns in wine choices, finding a positive willingness to pay (WTP) for certification systems guaranteeing production practices respectful of the environment, such as organic [22], low carbon emissions wines [23], biodynamic wines [24], and wines produced with biodiversity respectful practices [25,26]. On the contrary, to our knowledge, no previous study specifically addressed consumers’ preferences for wine in aluminium cans. However, some studies on consumers’ perception of alternative packaging have been conducted [1,21,27], and their results and insights represent the scientific basis of this research.

A consistent body of literature found that consumers prefer glass rather than aluminium cans, while alternative packagings are still considered of lesser quality [22,23,24,25,28,29,30,31]. Nesselhauf et al. [14] confirmed the preference for the more traditional packaging alternatives by investigating consumers’ acceptance for bottles with screw-cap closures, bag-in-box and StackTek^®^ (a single serving plastic package, StackTeck Systems Ltd., Brampton, ON, Canada), finding a higher acceptance for the glass bottle with screwcap than for any other alternative.

The reasons for the success of glass bottles over alternative (and more sustainable) packaging types are varied and rooted in the complexity of wine, whose numerous characteristics can influence the choice of the product, and in consumers’ attitudes, habits, preferences, and beliefs.

Although there is no literature specifically focusing on consumer preferences for canned wine, various information comes from alternative packaging literature. Barber et al. [27,32] found a positive relationship between the age of consumers and their acceptance to pay more for environment-friendly packaging, while other characteristics as the level of education and the place in which people live did not affect respondents’ choices.

Ferrara et al. [1] proposed a survey on consumers’ perception of alternative packaging, considering Bag in Box, Tetra Pack, and PET bottles, and found that some consumers would be willing to switch to more sustainable wine containers if informed that the wine quality does not change in alternative packaging, and that by using them the wine sustainability would improve. Moreover, the acceptance of the innovation increases with the increasing level of information about the innovative product [1,21]. Ferrara et al. [1] also found that consumers interested in alternative packaging are generally not strong drinkers and do not consider the features of the glass bottle in their buying choices.

Due to its complexity, wine can be described in terms of several features such as colour, glassware, branding, label design, closure type, and pricing, gaining the podium of the most studied beverage globally [33,34]. It has been proven that the colour of wine influences the tasting [35,36]; thus, a see-through packaging can be determinant in wine choices. The first impression of wine that consumers perceive is given by the colour, which can provide lots of information about the ageing and the climatic conditions of the specific vintage [37]. Furthermore, aluminium cans and all forms of alternative packaging, which are in many cases more sustainable than glass bottles, are generally perceived as unsuitable for containing a refined product, which matures over time, such as wine [13,14].

Moreover, complex emotional factors should be considered, among which the context in which wine is consumed, for which a packaging typology may be preferred to another [34], and specific behaviours and beliefs, supported by more or less founded reasons, that might affect consumers’ quality perception of the packaging options. For example, Piqueras-Fiszman and Spence [38] showed a positive relationship between increased wine bottle weight and wine price. Other suggestions are given by researchers focusing on the sound of closure, confirming a preference for cork compared to screwcap [37]. Moreover, Italian wine consumers are generally traditionalists and consider alternative packaging not suitable for wine products [1].

## 3. Materials and Methods

### 3.1. Data Collection and Survey Structure

The data collection for this research was performed with a survey posted on the free online survey administration platform “Google Forms” and disseminated through social networks (Facebook, Linkedin, and Twitter) on pages and groups dedicated to the world of wine.

Collecting data through a questionnaire distributed online using a convenience sample entails several advantages but also problems. Very high numbers of potential respondents can be reached in a short time and at significantly reduced costs, and the response rate is higher compared to more traditional forms of distribution [39,40]. On the other hand, the population to which online surveys are distributed cannot be described; the sample might not represent the population and respondents with biases may select themselves into the sample, jeopardising the reliability of the results. However, comparing online and mail survey administration modes in contingent valuation, Ryan et al. [40] found minimal differences in respondents’ declared WTP, concluding that internet panels generate valid and cost-effective results. Moreover, in this research, bias reduction strategies have been put in place in the form of a cheap-talk script, alerting respondents to tendencies to overstate values and reminding them of the importance of truthfully answering the questions [41,42].

Generic consumers of alcoholics were the main target of this research, but anyone could fill in the survey. This procedure has been chosen to reasonably select a sample of subjects not necessarily only composed of wine lovers and to collect information from a large number of voluntary respondents without the intermediation of an interviewer, thus avoiding any interviewer bias [39]. At the end of the first section, the question “do you drink wine?” identified respondents who do not drink wine. From an initial sample of 606 adult respondents, this procedure selected 551 participants for the complete survey (90.9%). Each participant took around 10 min to complete the survey. Data collection took place from October to November 2019.

A pilot questionnaire was developed and pre-tested on a sample of respondents and tuned up before distribution. The questionnaire included 24 Likert-scale questions and one open question, covering different topics. The choice of questions and variables to be included in the survey was preceded by a series of meetings with consumers, producers, and wine experts to identify the relevant aspects of canned wine consumption. The questionnaire was composed of four sections: a first introductory section explained the scope of the research as an analysis of the preferences of wine consumers, without providing information that could have guided participants’ responses, and explained how to fill in the survey. The second section dealt with alcohol consumption, wine preferences and characteristics consumers attribute to canned wine. Section 3 contained the contingent valuation question to elicit consumers’ WTP for a 25 cl aluminium can of wine and some follow up questions to better frame the motivations behind the answers. In the WTP estimate question, consumers were presented with a brief description of a 25 cl aluminium can containing Ribolla Gialla wine, a product much appreciated and widely known by Italian consumers, both in terms of prices and quality. To limit overestimates and unrealistic values, we have limited the choice of the WTP to 4 options of increasing price ranges.

Respondents who stated they were unwilling to buy the canned wine were asked to explain why and respondents with a positive WTP were asked to declare the main reasons for which they would buy it. The last section of the questionnaire addressed respondents’ demographics and general information. A final section of the questionnaire invited open comments, which were analysed qualitatively to aid the classification of the WTP motivations.

### 3.2. Contingent Valuation Econometric Model Specification

This research uses a contingent valuation approach to elicit consumers’ WTP for canned wine [43,44]. The contingent valuation method estimates the economic value of goods “without a market” through a direct survey that detects consumer preferences. It is based on the simulation of a hypothetical market and aims to estimate the WTP to improve the level of well-being or the willingness to accept to give it up. Over the years, this methodology has been applied in research on wine consumers in numerous different situations to estimate the value of different items such as organic wines [45], socially acceptable certified wines [46], sustainable wines [47], and the profitability of establishing local organic wine markets [48], among the others.

Zero Inflated Ordered Probit (ZIOP) was developed to model ordered response variables when the data exhibit a spike of zeros followed by a right-skewed continuous distribution of positive values [49,50]. This is common in several fields where data collection provides a status quo, neutral, or zero outcomes option [51]. For example, as explained by Harris and Zao [49], the question “how much would you pay for a can of wine” will probably collect a large number of answers equal to 0 €, or “I would not buy it”. If some of these zeros come from the so-called genuine non-participants, i.e., people who do not consider consumption in their decision-making process, others are from potential users, more responsive to standard consumer demand factors such as prices and income [49].

Whereas traditional Ordered Probit models fail at explaining data with an excess of zero observations, the ZIOP model is based on a two-stage decision process that perfectly suits this type of data. Respondents first decide whether to opt-in in a process (for example, buy or not buy a can of wine) and then, conditional on participating, decide the level of involvement (how much to pay for the canned wine), including the zero levels. The first decision is a binary choice and is modelled using a probit model, while the second is an ordered choice and is modelled through an ordered probit model [49]. ZIOP model has been widely used in studies on tobacco consumption [49], cannabis consumption [52], truck drivers’ violation frequency [53], sports participation [54], and health-related issues [55].

Let *s_j_* = 1 if the *j*^th^ individual is a participant or let *s_j_* = 0 otherwise. With the probit model, the probability of participation is given by Equation (1):

(1) $$Pr\left(s_{j}=1|z_{j}\right)=\Phi \left(z_{j}\gamma \right)\;$$

where *z_j_* is a vector of covariates that determines group membership, γ is a vector of coefficients that need to be estimated, and Φ (.) is the function of standard normal distribution. Next, conditioning on *s_j_* = 1, ordered probit model is used to model participation levels ${\tilde{y}}_{j}$; these levels may also include zeros. The corresponding probabilities are given by Equation (2):

(2) $$Pr\left({\tilde{y}}_{j}=h|s_{j}=1,x_{j}\right)=\Phi \left(\kappa_{h}-x_{j}\beta \right)-\Phi \left(\kappa_{h-1}-x_{j}\beta \right)h=0,1,\ldots ,H$$

where $k_{-1}=-\infty$, $k_{H}-1=\infty$ and *x_j_* is a vector of covariates that can differ from *z_j_*. *k_h_* are boundary parameters that need to be estimated in addition to the coefficients vector *β*. The intercept *β*_0_ is set equal to 0 in (2) for identification. *s_j_* and ${\tilde{y}}_{j}$ are both unobservable in terms of zeros. The observed response variable is $y_{j}=s_{j}{\tilde{y}}_{j}$. Thus, the zero outcome occurs when *s_j_* = 0 (non-participants) or when *s_j_* = 1 and ${\tilde{y}}_{j}$ = 0 (participant with zero activity). In order for *y_j_* to have a positive value, both conditions must be met that *s_j_* = 1 and ${\tilde{y}}_{j}$ > 0. The distribution of *Y* is given by Equation (3):

(3) $$\begin{array}{cc} Pr\left(Y\right) & =\{\begin{array}{c} Pr\left(y_{j}=0|z_{j},x_{j}\right)\\ Pr\left(y_{j}=h|z_{j},x_{j}\right) \end{array}h=1,2,\ldots ,H\\ & =\{\begin{array}{c} Pr\left(s_{j}=0|z_{j}\right)+Pr\left(s_{j}=1|z_{j}\right)Pr\left({\tilde{y}}_{j}=0|s_{j}=1,x_{j}\right)\\ Pr\left(s_{j}=1|z_{j}\right)Pr\left({\tilde{y}}_{j}=h|s_{j}=1,x_{j}\right) \end{array}h=1,2,\ldots ,H \end{array}$$

The occurrence of zero values is higher because it results from the sum of the probability of zero activity from the ordered probit model and the probability of non-participation from the probit model. Substituting Equations (1) and (2) in Equation (3), we obtain Equation (4):

(4) $$\begin{array}{c} Pr\left(Y\right)=\{\begin{array}{c} Pr\left(y_{j}=0|z_{j},x_{j}\right)\\ Pr\left(y_{j}=h|z_{j},x_{j}\right)\\ Pr\left(y_{j}=H|z_{j},x_{j}\right) \end{array}h=1,2,\ldots ,H-1 \end{array}$$

$$=\{\begin{array}{c} \left\{1-\Phi \left(z_{j}\gamma \right)\right\}+\Phi \left(z_{j}\gamma \right)\Phi \left(\kappa_{0}-x_{j}\beta \right)\\ \Phi \left(z_{j}\gamma \right)\left\{\Phi \left(\kappa_{h}-x_{j}\beta \right)-\Phi \left(\kappa_{h-1}-x_{j}\beta \right)\right\}\\ \Phi \left(z_{j}\gamma \right)\left\{1-\Phi \left(\kappa_{H-1}-x_{j}\beta \right)\right\} \end{array}h=1,2,\ldots ,H-1$$

The log-likelihood function is:

(5) $$\ln\;L\;=\;\sum_{j=1}^{N}\;w_{j}\;\sum_{h=0}^{H}\;I(y_{j}=h\;)\;ln\{Pr(y_{j}=h\;|\;z_{j},\;x_{j})\}$$

where *w_j_* is an optional weight for the *j*^th^ observation and

(6) $$I\left(y_{j}=h\right)=\{\begin{array}{cc} 1 & if\;y_{j}=h\\ 0 & otherwise \end{array}$$

### 3.3. Characteristics of the Sample

As shown in Table 1, respondents are adequately distributed across genders and age categories, with a slight overrepresentation of the respondents between 18 and 25 years old (32%). Furthermore, 45% of respondents declare an annual income below 35,000 €, probably because more than half of the sample is represented by young people under 36 years (57% of the total). At the same time, 37% of respondents have an annual income between 70,000 € and 100,000 €, probably related to the age class of 36–55, which typically stands for working people with families. In addition, most of the sample have a good level of education; around 96% have a diploma, and a significant part of the sample has a degree (63%).

Table 2 presents the questions and answers regarding habits and attitudes toward wine consumption of the sample, in the same order they were presented in the questionnaire. Over 70% of the sample spends less than twenty euros per week on wine consumption and drinks wine between one and three times a week. Just over a quarter of the sample drinks almost every day and spends over twenty euros a week on the purchase of wine. Respondents were asked to indicate whether, according to their habits, their personal consumption of wine takes place mainly in a private space such as their home rather than in moments of free time spent outside the home. About 38% of the sample stated that they do not have a strict prevalence of wine consumption at home rather than outside the home, while less than 25% said they consume wine mainly within their own home. Similarly, in terms of preferences for alcoholic beverages, over 53% of respondents state that they do not have a strict preference between wine and other alcoholic beverages such as beer or spirits, while just under 25% declare that they have a strict preference for wine. Additionally, 68% of respondents would expect to find white wine inside an aluminium can compared to 36% for red wine and 13% for rosé wine. Moreover, 79% of the respondents heard about canned wine for the first time during the survey. Finally, compared to placing the product inside the aisles of a supermarket, almost 70% of respondents would expect to find canned wine in the wine department, while only 30% would see them better among canned drinks.

## 4. Results

**Figure 1:**
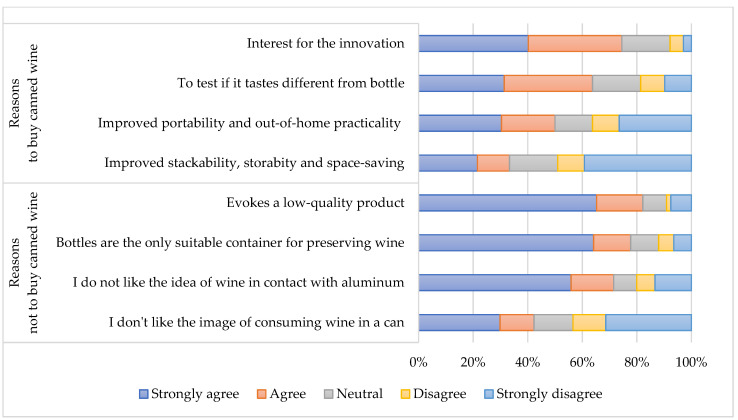
Main reasons related to the purchase (*n* = 102)/non-purchase (*n* = 449) of canned wine.

Only 19% of the sample declared to be interested in buying canned wine, while the majority expressed opposition. Depending on their inclination to buy or not to buy canned wine, respondents were also asked to justify their choice by indicating how much they agreed or disagreed with various statements that the literature analysis highlighted among the main drivers of choosing to buy or not wine in alternative and more sustainable packaging; the answers are represented in Figure 1.

The most mentioned reasons are the evocation of a low-quality product (83%) and the belief that glass bottles are the only container suitable for containing a delicate product such as wine (75%). A substantial part of the respondents does not believe that aluminium is a suitable material for the conservation of wine (77%), and in this regard, they have health-related and qualitative doubts. Only a minority of the sample (43%) manifested concerns about the aesthetic aspects and personal image that canned wine consumption could entail. Among the reasons for choosing to buy canned wine, the most mentioned relate to curiosity for a new product and the willing to taste any differences with bottled wine, while half of the sample mentioned reasons dealing with the higher practicality of aluminium cans especially in out of home situations. Only a small minority of the sample mentioned the better stackability and storability of cans compared to bottles.

Table 3 presents the results of the ZIOP regression. For both dependent and independent variables, the categories whose frequencies represent less than 10% of the sample were aggregated together to avoid bias in the results. The first two columns (Buy/Not buy) report the results of the binary choice between buying and not buying canned wine modelled using a probit model; marginal effects are included. The second column (WTP) reports the results referring only to the positive WTP declared in the contingent evaluation, modelled through an ordered probit model.

The variable Age is significant for the 36–55 and the 56–65 categories in both the models, being 18–25 the reference base. However, the effect is different in the two models: people older than 35 declare to be more interested in buying canned wine but are willing to pay less than younger respondents. On the contrary, respondents under 36 are less interested in buying canned wine, but they recognise it a higher price when they are willing to buy it. Nesselhauf et al. [21] investigated consumers’ preference for environmental-friendly wine packaging and found that the millennial generation is less interested in innovation in wine packaging than the baby boomers generation. Moreover, according to Castellini and Samoggia [56], young people are more interested in novelties and innovation when they are offered an attractive image and accompanied by information on the product: “they search for a supportive purchasing experience” (p. 137). Thus, no additional information about canned wine benefits was provided in this research, and young consumers may have been less willing to purchase it.

The variable “Income” is negatively correlated with the probability of buying canned wine, and the effect is more evident as the income increases. More in detail, individuals with an annual income from 35,000 € to 70,000 € are about 12% less likely than respondents with an annual income lower than 35,000 € to be interested in buying canned wine, up to 33% for people earning over 70,000 € per year. These results seem to highlight a greater interest in canned wine by consumers with modest incomes. Among respondents willing to purchase canned wine, those with a higher annual income have a higher WTP than people with an annual income lower than 35,000 €. Consumers with a higher income are generally willing to spend more on non-essential goods such as wine [25], but also food products with specific characteristics, such as organic and fair-trade products [57,58].

The variable “Weekly_frequency” is statistically significant, being the base level respondents who drink once a week. As the self-reported frequency of wine consumption increases, the likelihood of being willing to buy canned wine decreases: on average, individuals who drink 2 to 3 times a week are about 19% less likely to be interested in buying a can of wine than occasional drinkers. The effect is even more substantial for respondents who declare to drink almost every day and every day, who have a 41 % lower probability of being willing to pay for canned wine than occasional drinkers. Therefore, as the weekly frequency of consumption increases, the probability that individuals are willing to buy canned wine decreases. This is confirmed by Ferrara et al. [1], where people declaring to be interested in alternative packaging were not frequent drinkers. Moreover, aluminium cans are perceived by the most as containing a cheap product, more suited for fun and social events, as happens for beer, scarcely fit for everyday consumption. Therefore, assiduous consumers might be less interested in purchasing single-serve containers, while those who drink occasionally show greater interest in buying cans, which do not require the consumption of an entire bottle. This result highlights an interest in canned wine by people who are not frequent drinkers, suggesting that canned wine strategies promotion should consider atypical wine consumers, as it can be of interest to a vast public, not only habitual and high spending drinkers. The weekly spending budget dedicated to wine consumption does not affect consumers’ decision whether or not to buy canned wine. However, among respondents who are willing to buy canned wine, those with a low weekly budget for wine have a higher WTP than those with medium spending availability (10–20 €/week). Thus, aluminium packaging could meet the interest of those consumers who do not spend large sums to drink wine, both because it is perceived as cheaper than bottled wine and because the quantity in a can is more suitable for their modest wine consumption needs. As for the variable “Main_place_of_consumption”, people that usually drink wine out of their homes are 18% less likely to be willing to buy canned wine than home drinkers. As mentioned above, by choosing canned wine, people are not obliged to open an entire bottle to enjoy a glass of wine, and they can choose different wines with different courses during the same meal. People who declared to have already heard the term “canned wine” are about 48% more likely to be willing to purchase canned wine when compared to people that have never heard the term before the survey. Thus, consumers’ knowledge about the product is associated with a higher acceptance of aluminium cans as wine packaging [21]. In some cases, the degree of consumer knowledge impacts the acceptance of innovations. For example, Nesselhauf et al. [21] found that the lowest the consumer’s level of knowledge about the product, the higher the impact of consumer information on his acceptance. Likewise, Atkin et al. [20] confirmed the importance of information to encourage the acceptance of innovations. Consumers’ expectations of the type of product found in a can reveal that the respondents expecting to find red wine in a can (“Red”) are 24% more likely to buy canned wine than individuals expecting to find white wine. However, among consumers who are willing to buy wine in aluminium cans, respondents expecting red wine are willing to spend less.

## 5. Conclusions

Even though canned wine is not a recent innovation, wine producers have been significantly investing in introducing this packaging in the mainstream wine market only in recent times. Canned wine is still little known among wine consumers, and it will take some time and effort to popularise it. In this sense, it is not surprising that the first findings of this research indicate that less than 21% of the sample knew about canned wine before taking part in the survey, and only 19% would be willing to buy it. Aluminium cans are still associated with poor quality products in the collective imaginary, and most consumers are not interested in buying them, for example, high-income consumers or with a high weekly wine budget. However, the association between low-quality wines and aluminium cans only partially describes the current trend in the wine market. Whereas a consistent share of the wines sold in cans is of low quality, according to the most important wine tasting websites, several products are of good quality and, in some cases, outstanding [59].

Most of the sample (79%) heard about canned wine for the first time during the survey, suggesting that the diffusion of information about wine cans is probably still very limited. This is even more important because wine is a product whose quality cannot be established before having tasted it, so providing information can become the success point of product innovation in the wine market. According to our results, the lack of knowledge (and communication) about canned wine is one of the most limiting factors for a favourable attitude towards buying canned wine. People who have already heard the term “canned wine” are more willing to buy this product. In this research, we investigate the homegrown value consumers attribute to a hypothetical wine in a can, without providing them with additional information about the advantages, especially in terms of environmental impact, that this type of packaging could bring to the wine industry. According to a wide body of literature [21,60], information and extrinsic cues about innovative products can foster their acceptability, especially when supported by adequate marketing strategies and activities.

The frequency of wine consumption contributes to describing consumers’ purchasing profiles [1], and the results show that the consumers most interested in buying canned wine are not the most frequent drinkers but mostly the occasional ones. Most frequent drinkers are likely to have a series of habits and beliefs that make it more difficult for them to accept a new form of wine packaging as aluminium cans, and therefore other aspects should be leveraged to bring them closer to this type of product. In this sense, it is not obvious that canned wine would directly compete with the traditional wine in bottles. On the contrary, considering the massive global production, the emerging markets and consumers, the change in consumption habits, canned wines could address and interest different groups of drinkers on several different consumption occasions, increasing the opportunities for winemakers.

In line with the findings of this research, some concluding remarks can be outlined. Wine in aluminium cans is an emerging and promising solution to enhance the wine market, particularly towards specific consumer targets. For instance, young and occasional wine consumers might be of interest to wine enterprises, but the different potential targets are still to be thoroughly identified and understood. The number of structured researches on alternative wine packaging is still narrow, and a limited group of enterprises is systematically investing in marketing analysis. Studies and research will increase the growing interest in alternative and innovative wine packaging, and economic experiments, online surveys, and other investigations will provide outlooks on regional and global markets, consumers’ WTP, and marketing and communication strategies.

This research is not without limitations. First, selection bias could affect sample selection due to the type of distribution of the questionnaire, which makes proper randomisation unlikely to achieve. Thereby, the final sample might not represent the population intended to be analysed. Second, this research analysed consumers’ WTP using a hypothetical method, subject to hypothetical bias, leading respondents to report unrealistic behaviours or values in surveys or experimental research. Further research should inspect whether the results carry over to real-life environments and use incentive-compatible methodologies to assess whether hypothetical or other possible biases could skew the results. Moreover, future research steps should include analysing consumers’ market segmentation and possible marketing strategies for canned wine.

## Figures and Tables

**Table 1:** Socio-demographic characteristics of the sample, absolute numbers, and relative frequency.

Variables	Description	Frequency (*n*)	Percentage (%)
Age	18–25	179	32
	26–35	139	25
	36–55	142	25
	56 or more	91	18
Yearly income	Up to 35,000 €	247	45
	35,001–70,000 €	40	7
	70,001–100,000 €	205	37
	More than 100,000 €	59	11
Education	Middle school	21	4
	High school	183	33
	Degree	347	63
Gender	Male	261	47
	Female	290	53

**Table 2:** Habits and attitudes of wine consumption of the sample, absolute numbers, and relative frequency.

Variables	Description	Frequency (*n*)	Percentage (%)
What is your weekly budget for wine? (Weekly_budget)	Up to 10 €	231	43
	More than 10 to 20 €	164	30
	More than 20 to 30 €	104	19
	More than 30 €	52	9
With which weekly frequency do you usually drink wine? (Weekly_frequency)	Once a week	176	32
	2/3 times a week	223	40
	Almost everyday	117	21
	Everyday	35	6
Where do you usually drink? (Main_place_of_consumption)	Mainly out of home	207	38
	Mainly at home	134	24
	In both situations, no prevalence	210	38
Which is your favourite beverage? (Favorite_beverage)	Wine	130	24
	Others (Beer, spirits, cocktails)	128	23
	More than one, no strict preference	293	53
Have you ever heard the term “canned wine”? (Knows_canned_wine)	Yes	113	21
	No	438	79
Which wine typology do you expect to find in a can? (Expectations)	White	278	50
	Red	200	36
	Rosè	73	13
Where would you prefer to find canned wine in the supermarket aisles?	Canned drinks department	179	32
	Wine department	372	68
Would you be interested in buying an aluminium can of wine?	Yes	102	19
	No	449	81
Willingness to pay for canned wine (WTP)	0	449	81
	Up to 3 €	51	9.5
	Between 3 and 6 €	48	8.7
	More than 6 €	3	0.8

**Table 3:** Zero Inflated Ordered probit results.

Variables	Categories	Buy/Not Buy (Probit)		WTP (Ordered Probit)
		Coefficients (Standard Error)	Marginal Effect (Participation)	Coefficient (Standard Error)
Age	18–25	reference base		
	26–35	−0.132 (0.393)	−0.026	−0.343 (0.338)
	36–55	0.866 * (0.488)	0.174 *	−0.216
	56 or more	1.498 *** (1.129)	0.701 **	−1.836 *** (0.415)
Income	Up to 35,000 €	reference base		
	35,001–70,000 €	−0.576 (0.360)	−0.115 *	0.36 (0.251)
	Over 70,000 €	−1.654 *** (0.507)	−0.332 **	1.239 *** (0.454)
Education	Middle/High School	reference base		
	Degree	−0.845 ** (0.343)	0.169 **	0.322 (0.247)
Gender	Male	reference base		
	Female	0.504 (0.300)	0.101	−0.159 (0.230)
Weekly_budget	Up to 10 €	−0.361	0.163 *	0.793 *** (0.285)
	10–20 €	reference base		
	More than 20 €	0.091 (0.396)	−0.018	0.012 (0.320)
Weekly_frequency	Once a week	reference base		
	2/3 Times a week	−0.459	−0.188 *	0.768 *** (0.273)
	Almost/everyday	−2.064 *** (0.710)	−0.414 **	1.230 *** (0.404)
Main_place_of_consumption	Home	reference base		
	Out of home	−0.896 ** (0.441)	−0.180 **	0.485 (0.374)
	Both	0.091 (0.366)	0.018	−0.241 (0.277)
Favorite_beverage	Other beverages	reference base		
	Wine	−0.345 (0.460)	0.0691	0.103 (0.353)
	Wine and other beverages	−0.034 (0.374)	0.0069	0.475 (0.298)
Knows_canned_wine	No	reference base		
	Yes	2.410 *** (0.515)	0.480 ***	−0.45 (0.308)
Expectations	White	reference base		
	Red	1.094 *** (0.382)	0.220 **	−0.637 ** (0.285)
	Rosè	0.267 (0.448)	0.050	−0.499 (0.352)
Observations (*n*)	551			
Log likelihood model	−295.316			
AIC (df = 47)	668.631			

* *p* < 0.05; ** *p* < 0.01; *** *p* < 0.001.

## Data Availability

The data that support the findings of this study are available from the corresponding author, [G.R.], upon reasonable request.
